# Sphenoid sinus pseudoaneurysm with carotid cavernous fistula presenting with delayed subarachnoid hemorrhage: A case report

**DOI:** 10.1097/MD.0000000000026383

**Published:** 2021-06-18

**Authors:** Min Jai Cho, Kyung Sik Yi, Chi-Hoon Choi, Kyu Sun Yum, Sang-Hoon Cha, Yook Kim, Jisun Lee

**Affiliations:** a Department of Radiology; b Department of Neurosurgery; c Department of Neurology; d College of Medicine and Medical Research Institute, Chungbuk National University, Cheongju, Republic of Korea.

**Keywords:** carotid-cavernous fistula, embolization, head trauma, sphenoid sinus pseudoaneurysm, subarachnoid hemorrhage

## Abstract

**Rationale:**

Sphenoid sinus pseudoaneurysm arising from the cavernous segment of the internal carotid artery (ICA) caused by traumatic vessel injury is rare, and rarer is a concomitant carotid-cavernous fistula (CCF). In particular, delayed subarachnoid hemorrhage (SAH) due to pseudoaneurysm rupture has not been reported to-date in literature. Here, we report a case of sphenoid sinus pseudoaneurysm with CCF presenting with delayed SAH.

**Patient concerns:**

A 73-year-old man presented with traumatic brain injury due to motorcycle accident.

**Diagnoses:**

Twenty-four days after admission, the patient's neurological status suddenly deteriorated. Brain computed tomography (CT) showed acute SAH along interhemispheric cisterns and suprasellar intracerebral hematoma. Brain CT angiography and digital subtraction angiography revealed giant sphenoid sinus pseudoaneurysm with CCF and the daughter sac of the pseudoaneurysm extended to the intracranial part via fracture in the superior wall of the sphenoid sinus.

**Interventions:**

As the sphenoid sinus pseudoaneurysm and CCF shared one rupture point, endovascular treatment with intraarterial approach using coil and liquid embolic material by balloon assisted technique was performed simultaneously.

**Outcomes:**

The origin of the pseudoaneurysmal sac and CCF was sufficiently blocked after injection of liquid embolic material and the lesions completely resolved immediately after endovascular treatment.

**Lessons:**

Sphenoid sinus pseudoaneurysm and CCF rarely occur following head trauma through a series of processes involving fracture of the lateral wall of the sphenoid sinus and ICA cavernous segment injury. Sphenoid sinus pseudoaneurysm may present as SAH through intracranial rupture with concomitant superior wall fracture of the sphenoid sinus. Therefore, early diagnosis using CT or magnetic resonance angiography and appropriate treatment through understanding the disease mechanism is necessary.

## 1 Introduction

Pseudoaneurysm in the sphenoid sinus caused by internal carotid artery (ICA) injury is a very rare complication of head trauma.^[1,2]^ It originates from the cavernous segment of ICA and located in the sphenoid sinus. Therefore, the main presenting symptoms are either massive epistaxis after aneurysmal rupture along the sphenoethmoidal recess – nasal cavity pathway or ophthalmic symptom caused by the mass effect.^[1,3]^ Since it occurs outside the dura, the chances of subarachnoid hemorrhage (SAH) from pseudoaneurysm rupture is extremely rare and to-date, has not been reported in literature. Additionally, though sphenoid sinus pseudoaneurysm is caused by an injury to the cavernous segment of the ICA, concomitant carotid cavernous fistula (CCF) has rarely been reported.^[4,5]^ Here, we report a case of sphenoid sinus pseudoaneurysm with CCF presenting with delayed SAH.

## 2 Case report

### 2.1 Patient information and clinical findings

**Figure 1:**
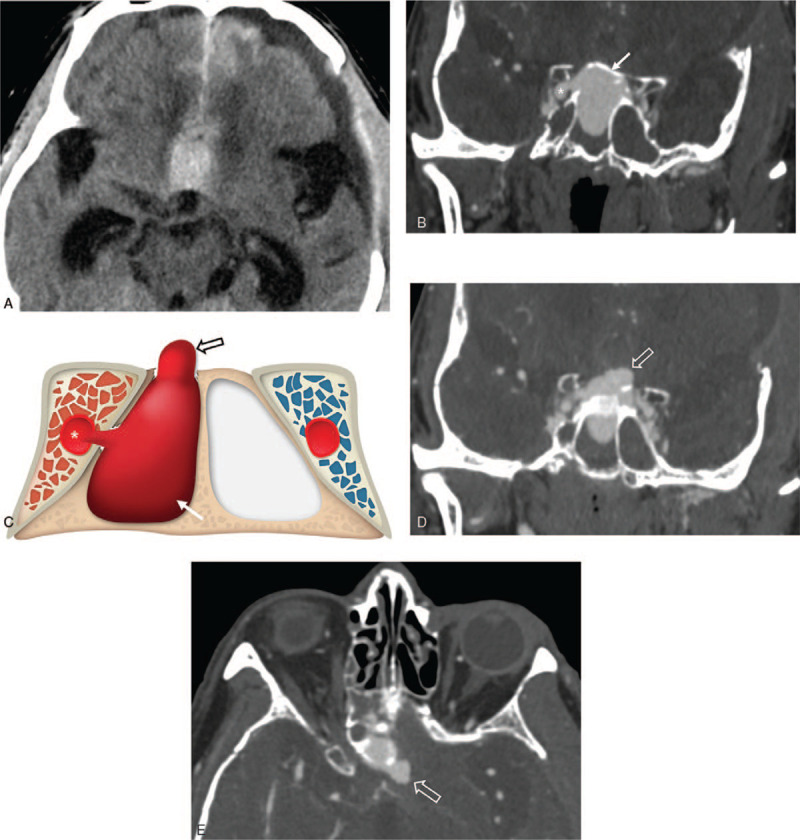
CT angiography and schematic illustration. (A) Brain CT showing suprasellar intracerebral hematoma and subarachnoid hemorrhage in the interhemispheric cisterns. Coronal CT angiography (B) and schematic illustration (C) showing giant sphenoid sinus pseudoaneurysm (arrow) originating from the cavernous segment of the right internal carotid artery (asterisk). The daughter sac (open arrow) of the pseudoaneurysm protruded to the intracranial part through the fracture defect in the superior wall of the sphenoid sinus as seen in the coronal (D) and axial (E) images. CT, computed tomography.

A 73-year-old man presented with traumatic brain injury due to motorcycle accident. The patient had no relevant past medical history. In the emergency room, initial Glasgow coma scale (GCS) score was 4 and his pupils were bilaterally fixed. Brain computed tomography (CT) scan showed diffuse SAH and acute subdural hematoma (SDH) in the left convexity, due to which 10 mm midline shift was observed. Cranial CT revealed multiple skull base fractures and ethmoid bone and sphenoid bone fractures. Emergency decompressive craniectomy and SDH removal were performed, after which, the patient was taken to the intensive care unit. The patient was sedated with midazolam to decrease brain metabolism and control intracranial pressure. Fourteen days after decompression, the patient regained consciousness enough for spontaneous eye opening and voluntary movement. On the 24th day, sudden neurological deterioration occurred. Follow-up brain CT revealed suprasellar intracerebral hematoma and SAH in the interhemispheric cisterns (Fig. 1A). Brain CT angiography revealed a giant sphenoid sinus pseudoaneurysm (Fig. 1B and C) that extended intracranially through concomitant superior wall fracture of the sphenoid sinus (Fig. 1D and E). Transfemoral cerebral angiography revealed CCF concomitant with the sphenoid sinus pseudoaneurysm, and a daughter sac, directed intracranially, was also detected (Fig. 3A). As the traumatic CCF and sphenoid sinus pseudoaneurysm extending intracranially had caused SAH, emergent embolization was performed.

### 2.2 Therapeutic interventions

The procedure was performed with a transfemoral arterial approach under general anesthesia. An 8-French shuttle sheath was advanced into the right ICA, placing the tip of the guiding catheter in the proximal ICA with a continuous flushing system. An SL-10 microcatheter with a 0.014-inch microguidewire (Synchro 14, Stryker, Kalamazoo, MI, USA) was advanced into the sphenoid sinus pseudoaneurysm. The triple-microcatheter technique was employed to occlude the pseudoaneurysm using multiple detachable coils (Fig. 3B). To embolize the residual CCF and pseudoaneurysm, Scepter balloon catheter (4 mm × 10 mm, Scepter C, MicroVention, Tustin, CA, USA) was deployed at the cavernous segment of ICA to prevent regurgitation to the ICA. Protecting the distal ICA with balloon microcatheter, intraarterial liquid embolic precipitating hydrophobic injectable liquid (PHIL, MicroVention, Tustin, CA, USA) was injected into the sphenoid sinus pseudoaneurysm and CCF (Fig. 3C). After injecting PHIL, the pseudoaneurysm and CCF were completely occluded and post-treatment ICA angiography revealed no visible sphenoid sinus pseudoaneurysm or shunt flow (Fig. 3E).

### 2.3 Outcomes

Seven days after embolization, GCS score of the patient recovered to 7 and pupillary responses were prompt. Follow-up brain CT angiography 14 days after the procedure showed no visible sphenoid sinus pseudoaneurysm or CCF and intact flow in the right ICA. The patient began spontaneous movement and eye opening, and was transferred to rehabilitation hospital 6 weeks after the intervention.

### 2.4 Ethical considerations

The institutional review board of approved the retrospective review of the medical records of the patient and waived the requirement for informed consent.

## 3 Discussion

**Figure 2:**
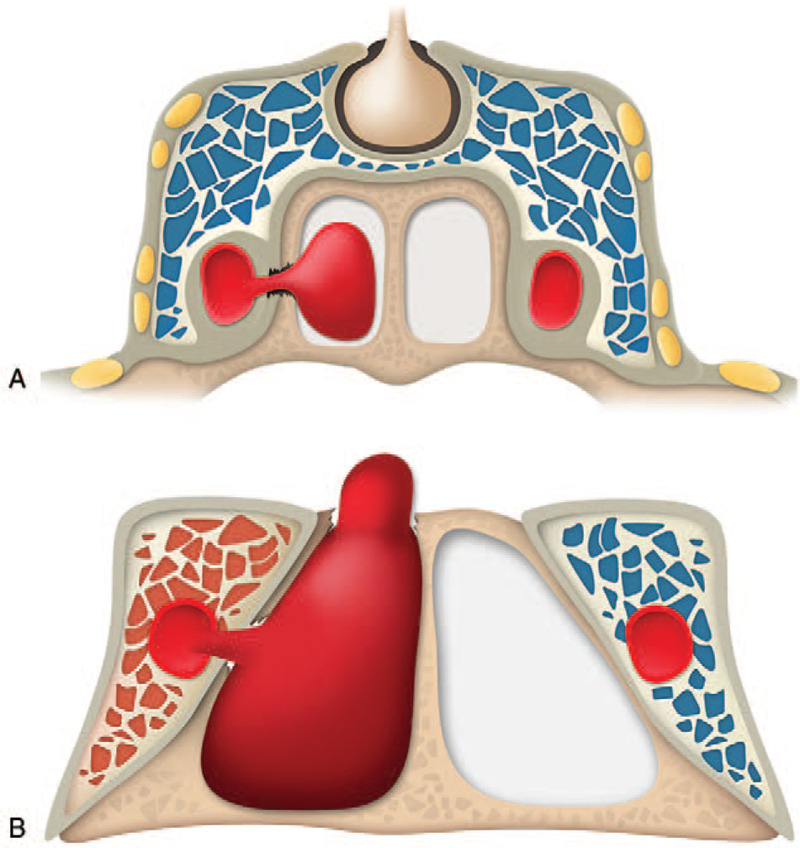
Schematic illustrations of types of sphenoid sinus pseudoaneurysms according to the presence of carotid-cavernous fistula. (A) Type 1: Isolated sphenoid sinus pseudoaneurysm. Sphenoid sinus pseudoaneurysm occurs alone due to damage to the point of contact between ICA and the lateral wall of the sphenoid sinus. (B) Type 2: Sphenoid sinus pseudoaneurysm concomitant with carotid-cavernous fistula. Injury to the medial wall of the ICA injury occurs where it is surrounded by the cavernous sinus, which spreads through the fractured lateral wall of the adjacent sphenoid sinus.

The carotid cavernous fistula is a well-known disease entity caused by vascular injury of the cavernous segment of ICA due to head trauma, and its incidence accounts for 0.2 to 0.3% of total traumatic head injury.^[6]^ Sphenoid sinus pseudoaneurysm is another disease entity caused by damage to the cavernous segment of the ICA, which is rare compared to CCF, and cases with concomitant CCF have rarely been reported.^[1,2,7–9]^ Theoretically, since the segment of ICA is located in the cavernous sinus, it is difficult to understand that CCF is not accompanied with pseudoaneurysm due to vessel damage. However, anatomically, a part of the cavernous segment of the ICA contacts the lateral wall of the sphenoid sinus to form the carotid sulcus, and the boundary is separated by bones with a thickness of less than 0.5 mm in about 88% of cadaver specimens.^[10]^ Certainly, not all parts of the cavernous segment are nested into the carotid sulcus, and there are areas that are completely surrounded by the cavernous sinus.^[10,11]^ Therefore, it is considered that two types of sphenoid sinus pseudoaneurysms occur, with and without CCF, depending on the site of the injury. In the case the injury in an area where the ICA and sphenoid sinus are in contact, sphenoid sinus pseudoaneurysm occurs without CCF. However, in the present case, it is presumed that the medial wall of the ICA was injured which spread through the fractured lateral wall of the adjacent sphenoid sinus, resulting in CCF with sphenoid sinus pseudoaneurysm (Fig. 2).^[5]^

**Figure 3:**
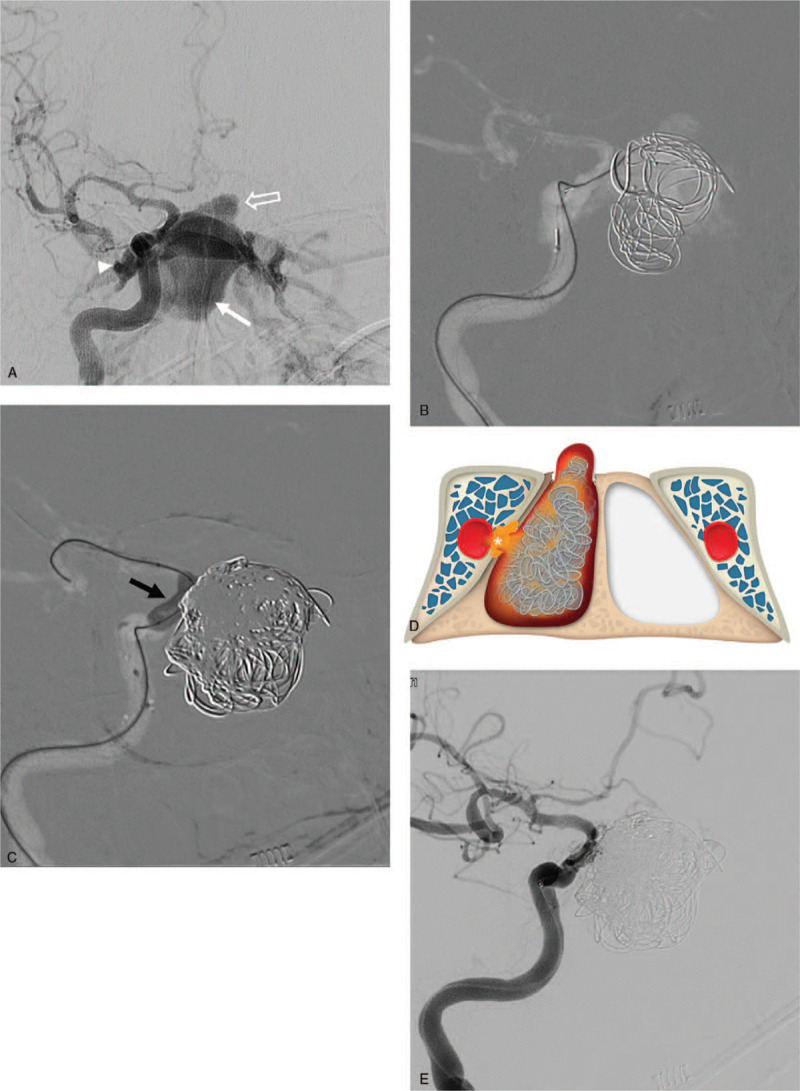
Cerebral angiography and endovascular treatment for sphenoid sinus pseudoaneurysm and carotid-cavernous fistula. (A) ICA angiography images showing sphenoid sinus pseudoaneurysm (arrow) and concomitant CCF (arrowhead), and daughter sac (open arrow) of the pseudoaneurysm found in intracranial direction. (B) Coil embolization was first performed through an intraarterial approach for the giant pseudoaneurysm. (C) After partial embolization of the aneurysmal sac using coils, intraarterial liquid embolic material containing precipitating hydrophobic injectable liquid (PHIL) was injected while protecting the ICA with a compliant balloon microcatheter (black arrow). (D) Schematic illustration of endovascular technique of embolization of the sphenoid sinus pseudoaneurysm and CCF. As the lesions shared one rupture point it is important to sufficiently block the origin of the rupture point (asterisk). (E) Post-embolization angiography showing the resolution of aneurysmal sac and CCF, and improved intracranial blood flow. ICA, internal carotid artery; CCF, carotid-cavernous fistula.

Sphenoid sinus pseudoaneurysm have mostly been reported as a direct connection from the cavernous segment of the ICA to the sphenoid sinus.^[2,3,7]^ Rupture of pseudoaneurysm reportedly causes recurrent or massive epistaxis, to the nasal cavity through the sphenoethomoidal recess, which is route drainage of the sphenoid sinus. Moreover, extrinsic compression by the mass of the pseudoaneurysm causes ophthalmic disorders such as monocular or binocular blindness.^[1]^ When CCF is concomitant with sphenoid sinus pseudoaneurysm, symptoms of venous engorgement such as exophthalmos, bruit, chemosis, and vision impairment may be part of clinical manifestations of the condition. However, poor state of consciousness of the patient following severe head trauma may prevent the detection of CCF symptoms, like the present case.^[12]^ In our case, delayed SAH occurred due to intracranial rupture of pseudoaneurysm, which has not been reported to-date in literature. Theoretically, sphenoid sinus pseudoaneurysm arising from the cavernous segment of the ICA is extradural and does not induce SAH. However, in our case, fracture of the superior wall of the sphenoid sinus also occurred, and the pseudoaneurysm that filled the sphenoid sinus formed a daughter sac towards the fracture defect (Fig. 1D and E). Therefore, if sphenoid sinus fracture follows head trauma, appropriate imaging investigations are required, since it is difficult to differentiate between sphenoid sinus pseudoaneurysm and sphenoid sinus hematoma with CT, and there is chance of misdiagnosis. Therefore, investigation with CT angiography, MRI, or MR angiography are necessary for early diagnosis.^[1]^

CCF and sphenoid sinus pseudoaneurysm are commonly treated with endovascular surgery.^[1]^ Sphenoid sinus pseudoaneurysm may be treated with coils or covered stent and detachable balloon through an intraarterial approach.^[13]^ However, pseudoaneurysm with CCF requires more complicated treatment as both need to be resolved. Since CCF will not resolve by embolization of aneurysmal sac alone, additional embolization using an intravenous or intraarterial approach is necessary to treat CCF. Through intraarterial approach, embolization may be performed by separately selecting pseudoaneurysm and cavernous sinus. However, since they share one rupture point as with the present case, simultaneous intervention may be considered if the ICA at the origin of the pseudoaneurysmal sac and CCF is sufficiently blocked (Fig. 3D). In our case, the pseudoaneurysmal sac was partially embolized with coils, and the remaining pseudoaneurysm and CCF were additionally treated by liquid embolic material injection while protecting the injured site of the ICA with a balloon microcatheter.

## 4 Conclusion

Sphenoid sinus pseudoaneurysm and CCF are rarely seen with concurrent injury of the cavernous segment of the ICA and the sphenoid sinus. In addition, SAH may occur due to pseudoaneurysm rupture through a fracture defect in the superior wall of the sphenoid sinus. Early diagnosis and prompt treatment is possible through in-depth understanding of the disease mechanism.

## Author contributions

**Conceptualization:** Min Jai Cho, Chi-Hoon Choi, Kyu Sun Yum, Sang-Hoon Cha, Kyung Sik Yi.

**Data curation:** Min Jai Cho, Yook Kim, Kyung Sik Yi.

**Formal analysis:** Min Jai Cho, Jisun Lee, Kyung Sik Yi.

**Supervision:** Chi-Hoon Choi, Kyu Sun Yum, Sang-Hoon Cha.

**Writing – original draft:** Min Jai Cho, Kyung Sik Yi.

**Writing – review & editing:** Min Jai Cho, Kyung Sik Yi.
